# Optimization of post-ripening treatments for efficient germination of early-harvested winter wheat seeds under speed breeding

**DOI:** 10.3389/fpls.2026.1940423

**Published:** 2026-09-14

**Authors:** Xiaoli Yu, Ming Zhao, Shengliang Cao, Tianying Yu, Haijing Xin, Xingxing Peng, Xiaoyun Du, Lingyun Li, Yanbo Wang, Xuechen Zhao, Lili Zhang, Mingxuan Li, Weihua Xu, Yukang Yang, Zhaofeng Li, Xinyu Wang, Jianpeng Zheng, Xiaohui Sun

**Affiliations:** 1 Yantai Key Laboratory of Wheat Resources Precise Evaluation and Biological Breeding, Yantai Academy of Agricultural Sciences, Yantai, China; 2 College of Life Sciences, Yantai University, Yantai, China; 3 College of Agriculture, Shihezi University, Shihezi, China

**Keywords:** early harvest, efficiency index, orthogonal experiment, seed vigor, single seed descent, speed breeding, winter wheat

## Abstract

Speed breeding (SB) can substantially shorten crop generation cycles; however, premature harvest often results in low germination percentages due to insufficient physiological maturity. In this study, the winter wheat cultivar ‘Yannong 301’ was used to optimize post-harvest treatments for early-harvested seeds. Single-factor experiments showed that 20–25 days after anthesis (DAA) represented a critical window during which germination percentage surged from 34.63% to 64.67% (*P*< 0.01). The optimal drying temperature was 40 °C in the drying temperature experiment. In the drying duration experiment using seeds harvested at 30 DAA, germination percentage reached 80.00% after 5 d of drying at 40 °C. Additionally, in the moist pre-treatment experiment using seeds harvested at 30 DAA, a 4-d treatment increased germination percentage to 95.94%. Orthogonal experiment further revealed that grain-filling duration after anthesis was the most important factor affecting germination percentage, with 23 days after anthesis (DAA) as the earliest suitable harvest time. The final optimized protocol was: harvest at 23 DAA + drying at 40 °C for 2 d + moist pre-treatment at 4 °C for 2 d + 1% H_2_O_2_ soaking for 1 d, achieving 97.06% germination within 35 d from anthesis to the end of the germination test. This protocol represents a practical optimum under the tested conditions. Validation across 10 winter wheat cultivars showed that germination percentages ranged from 80.57% to 95.69%, significantly outperforming untreated controls. The protocol also achieved a line-based seedling emergence rate of 97.5% (195/200) in a single seed descent (SSD) population, indicating its potential to support efficient single-seed descent advancement. This study identified grain-filling duration after anthesis as the key limiting factor for seed germination of winter wheat under SB, and established an efficient post-ripening treatment workflow that achieved high germination percentage and stable performance, providing critical technical support for large-scale breeding population construction and efficient multi-generation cycling.

## 1 Introduction

Global climate change and population growth pose severe challenges to food security, making it crucial to accelerate the breeding of staple crops such as wheat (Watson et al., 2018; Imam et al., 2024). However, conventional breeding typically requires 8–12 years to develop a new cultivar (Wanga et al., 2021; Samantara et al., 2022), a timeline that is insufficient to meet the increasingly urgent demand for yield improvement. SB technology, by optimizing photoperiod and temperature regimes, can dramatically shorten crop generation cycles to 4–6 generations per year, offering revolutionary potential for accelerating genetic improvement (Ding et al., 2005; Hickey et al., 2017; Ghosh et al., 2018; Watson et al., 2018; Voss-Fels et al., 2019; Samantara et al., 2022; Potts et al., 2023; Schoen et al., 2023).

In SB practice, the requirements for seed germination quality vary with the research objectives. Some studies focus on single seed descent (SSD) as the primary goal, requiring only a few seeds to survive for generation advancement (Watson et al., 2018; González‐Barrios et al., 2020; Gopinathan et al., 2025). However, when constructing genetic populations, conducting precise phenotyping, or reserving sufficient seeds for subsequent generations, large quantities of high-vigor, uniformly germinating seeds are required. Low and uneven germination can lead to insufficient population size, non-uniform seedling growth, increased workload due to repeated sowing, and even compromised accuracy of genetic selection (Finch-Savage and Bassel, 2015; Ghadirnezhad Shiade et al., 2024). Therefore, establishing a post-ripening workflow that achieves high germination percentage, consistent germination, and high time efficiency is of great practical significance for expanding the application potential of SB.

Seed vigor is significantly affected by developmental maturity and dormancy characteristics (Cheng et al., 2019; Shen et al., 2024). Under conventional conditions, seeds must undergo sufficient dry matter accumulation, desiccation, and after-ripening on the mother plant to achieve high germination capacity (Aihua et al., 2013; Du et al., 2015). Premature harvest interrupts this process, leaving seeds in a dormant or low-vigor state (Gao et al., 2013; Chen et al., 2019; Bomireddy et al., 2024). Watson et al. (2018) found that spring wheat seeds harvested prematurely at 14 days after anthesis (DAA) could achieve germination rates of 80%-100% after moist pre-treatment at low temperature, demonstrating the feasibility of combining early harvest with post-harvest treatments. However, existing studies have mainly focused on spring wheat and other spring-habit cereal crops; whether these parameters are applicable to winter wheat remains unclear. Our greenhouse pre-experiment revealed that the winter wheat cultivar ‘Yannong 301’ harvested before 15 DAA showed near-zero germination even after the same moist pre-treatment and hormone priming (data not shown), indicating that the germination potential of early-harvested seeds is highly dependent on genetic background and cultivation system, and that directly applying the existing parameters for spring wheat may lead to germination failure in winter wheat.

Various post-harvest treatment strategies have been employed to improve seed vigor. Dry after-ripening promotes internal metabolic conversion in seeds, and drying at 40 °C can better maintain the germination potential of rapeseed (Benyoucef and Panigrahi, 2025). Cold stratification (e.g., moist pre-treatment at low temperature) is a commonly used technique for breaking physiological seed dormancy (Matus-Cádiz and Hucl, 2003; Buitink and Leprince, 2008; Gao et al., 2013; Poff et al., 2016; Ali et al., 2022; Chen et al., 2022a) and has been proven effective in deeply dormant plants (Chen et al., 2020; Gao et al., 2021; Chen et al., 2022b) as well as in cereal crops under SB systems (Watson et al., 2018; González‐Barrios et al., 2020; Gopinathan et al., 2025). Exogenous hormones and reactive oxygen species are also important germination-promoting agents (Watson et al., 2018; Shah et al., 2023).

A preliminary comparison of pre-germination treatments showed that 1% hydrogen peroxide (H_2_O_2_) soaking for 24 h achieved a germination percentage of 95.33%, whereas GA_3_ at 30–60 mg/L produced 34%–58.67%, and the water control produced only 6.67% (**Supplementary Figure S1**). Therefore, 1% H_2_O_2_ soaking for 24 h was applied uniformly throughout this study. However, the above methods are mostly targeted at conventionally mature seeds. For winter wheat seeds with highly heterogeneous physiological maturity under SB systems, there is a lack of in-depth research on systematically optimizing drying and moist pre-treatment parameters. Moreover, how to jointly determine the earliest effective harvest node remains largely unknown. Thus, there is an urgent need to conduct systematic optimization of harvest time and post-harvest treatment parameters in a winter wheat SB system.

In this study, the winter wheat cultivar ‘Yannong 301’ was used as the experimental material, and an optimization strategy combining single-factor experiments and multi-factor orthogonal design was adopted. First, the effects of grain-filling duration, drying temperature, drying duration, and moist pre-treatment duration on seed vigor were systematically investigated through single-factor experiments to identify key limiting factors. Then, orthogonal experiment was employed to analyze the main effects of grain-filling duration after anthesis, drying duration at 40 °C, and moist pre-treatment duration at 4 °C, so as to determine the optimal level combination. This study innovatively introduced the “Efficiency Index” (EI = germination percentage/total duration) as a comprehensive evaluation metric, aiming to screen for a post-harvest treatment protocol that maximizes germination percentage while balancing time efficiency. On this basis, the selected optimal protocol was validated for its applicability in ten winter wheat cultivars with different genetic backgrounds and in an SSD population consisting of 200 lines. The results will provide key technical parameters for improving the large-scale production of high-quality seeds in SB systems, thereby contributing to the enhancement of wheat breeding efficiency.

## 2 Materials and methods

### 2.1 Plant materials and growth conditions

Main experimental material: Winter wheat (*Triticum aestivum* L.) cultivar ‘Yannong 301’ (YN301).

Multi-cultivar validation materials: Ten winter wheat cultivars/accessions with distinct genetic backgrounds were selected, including YN301 (control), Yannong 1212 (YN1212), Kenong 9204 (KN9204), Jimai 22 (JM22), Jimai 57 (JM57), Zhengmai 379 (ZM379), Nongda 753 (ND753), Xinong 511 (XN511), Xindong 33 (XD33), and Xinmai 88 (XM88) (see **Supplementary Table S1** for detailed information). All cultivars/accessions were obtained from the germplasm collection maintained at Yantai Academy of Agricultural Sciences.

SSD population material: An F_3_ recombinant inbred line population (200 lines in total) derived from the cross ‘Zhongke 311’ × ‘Xinong 511’ was used as the experimental material. Plants of the SSD population were cultivated in cylindrical growth tubes (4 cm in diameter, 20 cm in height).

Growth conditions: The experiment was conducted in an artificial climate growth chamber. Seeds were surface-sterilized (1% sodium hypochlorite (NaClO), 10 min) and rinsed with deionized water, then soaked in 1% H_2_O_2_ solution for 1 d to promote germination (Wu, 1982; Chu et al., 2022). After radicle emergence, seeds were sown in 128-cell plug trays (substrate: peat: vermiculite: perlite = 3:1:1). At the one-leaf stage, seedlings were transferred to a vernalization chamber (3 ± 1 °C, 16 h light/8 h dark, photosynthetic photon flux density (PPFD) 150 ± 25 μmol·m^-^²·s^-^¹) for 30 d (Allard et al., 2011). After vernalization, seedlings were transplanted into trapezoidal pots (upper diameter 18.5 cm × 18.5 cm, height 18 cm, 8 plants per pot) (plants of the SSD population were transplanted into cylindrical growth tubes of 4 cm in diameter and 20 cm in height, one plant per tube) and directly grown under SB conditions: photoperiod 22 h light/2 h dark, temperature 22 ± 1 °C, relative humidity 60%-70%. The light source was LED red-yellow-blue tri-color plant growth lamps (ZK3-TB14-VE03/A, Zhongke San’an, Fujian, China), with a canopy PPFD of 250 ± 25 μmol·m^-^²·s^-^¹. The same photoperiod (22 h light/2 h dark), temperature (22 ± 1 °C), relative humidity (60%–70%), and PPFD (250 ± 25 μmol·m^-^²·s^-^¹) were maintained throughout grain filling until harvest, ensuring consistent environmental conditions during seed development and maturation.

### 2.2 Germination assay

After corresponding treatments, seeds were placed in Petri dishes lined with filter paper and subjected to standard germination tests (in accordance with ISTA International Rules for Seed Testing) in an artificial climate chamber (FLI450, Faithful, Hebei, China; 20 ± 1 °C, 12 h light/12 h dark). Germination [defined as radicle protrusion through the seed coat and elongation to ≥ 2 mm (Bewley, 1997)] was recorded daily for 7 d. The following indices were calculated:


Germination energy (GE, %)=(Number of germinated seeds in the first 3 d/Total number of tested seeds)×100%

(Zhang et al., 2020)


Germination percentage (GP, %)=(Number of germinated seeds in 7 d/Total number of tested seeds) × 100%

(Zhang et al., 2020).


Germination index (GI):GI=Σ(G/Dt) 

where *Gt* is the number of germinated seeds on day t, and Dt is the corresponding germination day (Wang et al., 2019).

Vigor index (*VI*): *VI* = *GI* × *S*, where S is the average fresh weight (g/plant) of normal seedlings on day 7 (Wang et al., 2019).

Each treatment group contained 30–100 seeds, with the exact number per replicate reported in the corresponding **Supplementary Tables** (**Supplementary Tables S2–S10**). For each treatment, three biological replicates were established. Each biological replicate consisted of seeds pooled from five main-stem spikes, with each spike collected from a different plant grown in a separate pot. The three replicates were obtained from independent plant groups maintained under identical growth conditions. Germination tests for each replicate were conducted in separate Petri dishes.

### 2.3 Experimental design

#### 2.3.1 Single-factor experimental design

##### 2.3.1.1 Effect of harvest time on seed germination

The day on which the central spikelets of the main-stem spike flowered (anthers visible) was designated as the flowering date (0 DAA). Wheat spikes were harvested at 15, 20, 25, 30, and 35 DAA, respectively. Each time point had three biological replicates, with five main-stem spikes randomly selected per replicate. Spikes were uniformly dried in an electric forced-air drying oven (DFA-7000, Beijing Zhongxingweiye, China) at 40 °C for 5 d (pre-experiments indicated that seeds could be fully dried under this condition), then threshed. Hundred-grain weight was recorded, and standard germination tests were conducted immediately. Before standard germination tests, seeds were soaked in 1% H_2_O_2_ for 24 h at room temperature (22 ± 1 °C).

##### 2.3.1.2 Effect of drying temperature on seed germination

Seeds harvested at 30 DAA were threshed, and uniform seeds were selected. They were dried at 30, 35, 40, 45, 50, 55, and 60 °C in an electric forced-air drying oven (DFA-7000, Beijing Zhongxingweiye, China) for 3 d, with room temperature (22 ± 1 °C) air-drying for 3 d as the control. Standard germination tests were conducted after drying. Before standard germination tests, seeds were soaked in 1% H_2_O_2_ for 24 h at room temperature (22 ± 1 °C).

##### 2.3.1.3 Effect of drying duration at 40 °C on seed germination

Seeds harvested at 30 DAA were dried at 40 °C for 0, 0.5, 1, 1.5, 2, 2.5, 3, 3.5, 4, 4.5, 5, and 6 d. Seed weight loss was measured at each time point.


$$\text{Weight}\;\text{Loss}\;\left(\%\right)=(W_{0}-Wt)/W_{0}\times \;100\%$$

(Gebre et al., 2024)

where *W_0_* is the initial sample weight, and *Wt* is the sample weight after drying for t days.

Standard germination tests were conducted at the respective drying time points (0, 0.5, 1, 1.5, 2, 3, 4, 5, and 6 d). Before standard germination tests, seeds were soaked in 1% H_2_O_2_ for 24 h at room temperature (22 ± 1 °C).

##### 2.3.1.4 Effect of moist pre-treatment duration at 4 °C on seed germination

Seeds harvested at 30 DAA were used directly without prior drying. Seeds were placed in Petri dishes lined with filter paper, and sterile water was added (water level approximately 1/2-2/3 of seed height). They were treated in darkness at 4 °C for 1, 2, 3, and 4 d, with seeds that did not receive 4 °C moist pre-treatment and were directly subjected to germination tests serving as the control. Standard germination tests were conducted after treatment. Before standard germination tests, seeds were soaked in 1% H_2_O_2_ for 24 h at room temperature (22 ± 1 °C).

#### 2.3.2 Multi-factor orthogonal optimization experiment

To systematically investigate the effects of grain-filling duration after anthesis (A), drying duration at 40 °C (B), and moist pre-treatment duration at 4 °C (C) on seed germination, an L_16_(4³) orthogonal array was used for experimental design. Each of the three factors had four levels: A_1_-A_4_ corresponded to harvest at 17, 19, 21, and 23 DAA, respectively; B_1_-B_4_ corresponded to drying at 40 °C for 0, 1, 2, and 3 d, respectively; C_1_-C_4_ corresponded to moist pre-treatment at 4 °C for 0, 1, 2, and 3 d, respectively.

The experiment comprised 16 treatment combinations, each with three biological replicates, totaling 48 experimental units. All treatments followed a unified procedure: spikes were harvested at the designed DAA, dried at 40 °C for the specified number of days, and threshed; seeds were placed in Petri dishes lined with filter paper and subjected to moist pre-treatment at 4 °C for the designed number of days; after soaking in 1% H_2_O_2_ for 24 h, seeds were cultured under standard germination conditions, and germination percentage was recorded on day 7. The orthogonal experimental design scheme is presented in **Table 1**.

**Table 1:** Orthogonal experimental design [L_16_(4³)].

Run no.	A: Days after anthesis (d)	B: Drying duration at 40 °C (d)	C: Moist pre-treatment at 4 °C	Combination code
1	17	0	0	A_1_B_1_C_1_
2	17	1	1	A_1_B_2_C_2_
3	17	2	2	A_1_B_3_C_3_
4	17	3	3	A_1_B_4_C_4_
5	19	0	1	A_2_B_1_C_2_
6	19	1	0	A_2_B_2_C_1_
7	19	2	3	A_2_B_3_C_4_
8	19	3	2	A_2_B_4_C_3_
9	21	0	2	A_3_B_1_C_3_
10	21	1	3	A_3_B_2_C_4_
11	21	2	0	A_3_B_3_C_1_
12	21	3	1	A_3_B_4_C_2_
13	23	0	3	A_4_B_1_C_4_
14	23	1	2	A_4_B_2_C_3_
15	23	2	1	A_4_B_3_C_2_
16	23	3	0	A_4_B_4_C_1_

#### 2.3.3 Screening of optimal germination-promoting combinations based on harvest at 23 DAA

Based on the earliest effective harvest time identified among the tested treatments (23 DAA), six combinations of drying and moist pre-treatment durations were set up (**Table 2**) to further screen for a practical germination-promoting protocol that balances high germination percentage and short processing cycle. Each treatment had three replicates. All treatments included a 1% H_2_O_2_ soaking step for 1 d. The complete procedure for all treatments was: harvest at 23 DAA, dry at 40 °C for the specified number of days, thresh, apply moist pre-treatment at 4 °C for the specified number of days, soak in 1% H_2_O_2_ at room temperature (22 ± 1 °C) for 1 d, and germinate under standard conditions for 7 d.

**Table 2:** Design of germination-promoting treatment combinations for seeds harvested at 23 DAA.

Treatment	Combination code	Drying duration at 40 °C (d)	Moist pre-treatment at 4 °C (d)
1	A_4_B_2_C_4_	1	3
2	A_4_B_4_C_2_	3	1
3	A_4_B_3_C_3_	2	2
4	A_4_B_3_C_4_	2	3
5	A_4_B_4_C_3_	3	2
6	A_4_B_4_C_4_	3	3

Total time T (d) = 31 + B + C (see main text for calculation details).

To comprehensively evaluate the time efficiency of each treatment, an Efficiency Index (*EI*) was defined and calculated as *EI* = *GP*/*T*, where *GP* is the germination percentage (%), and T is the total number of days from the beginning of grain-filling to the end of the germination test. *T* = 23 (fixed grain-filling days) + B (drying days) + C (moist pre-treatment days) + 1 (H_2_O_2_ soaking day) + 7 (germination test days) = 31 + B + C. The EI value reflects the germination success rate obtained per unit time; a higher value indicates better overall efficiency.

#### 2.3.4 Multi-cultivar applicability validation of the optimized protocol

Ten winter wheat cultivars with different genetic backgrounds (among which YN301 was the cultivar used in the single-factor and orthogonal experiments of this study; **Supplementary Table S1**) were cultivated under identical SB conditions. For each cultivar, spikes were harvested at 23 DAA and treated according to the optimized protocol (designated as 23d-T): drying at 40 °C for 2 d, moist pre-treatment at 4 °C for 2 d, soaking in 1% H_2_O_2_ for 1 d, followed by a standard germination test as described in Section 2.2. Each cultivar and treatment had three replicates, and germination energy (day 3) and germination percentage (day 7) were recorded. Two controls were set up:

23d-CK: Spikes harvested at 23 DAA, air-dried at room temperature for 4 d (equal to the total duration of drying plus moist pre-treatment in the optimized protocol), then soaked in 1% H_2_O_2_ for 1 d before germination. This control was used to evaluate the gain in germination performance conferred by the optimized post-harvest treatment compared with simple air drying under the same grain-filling duration.
27d-CK: Spikes harvested at 27 DAA and directly soaked in 1% H_2_O_2_ for 1 d before germination. This control started germination at the same time as the optimized protocol: the total time from anthesis to the start of germination was 28 days for both treatments (optimized protocol: 23 d grain-filling + 2 d drying + 2 d moist pre-treatment + 1 d H_2_O_2_ soaking; 27d-CK: 27 d grain-filling + 1 d H_2_O_2_ soaking). This control served to assess the effect on seed germination percentage when the 4 days used for post-harvest treatment were instead allocated to continued grain-filling. It should be noted that this control did not include a drying step; therefore, the comparison between 27d-CK and 23d-T reflects the overall difference between two time-allocation strategies-early harvest plus post-harvest treatment versus extended grain-filling without post-harvest treatment-rather than the isolated effect of either grain-filling duration or post-harvest drying alone. This limitation is further addressed in the Discussion.

#### 2.3.5 Generation advancement validation of the optimized protocol in an SSD population

To validate the practical applicability of the optimized protocol in SSD breeding population, an F_3_ recombinant inbred line population (200 lines in total) derived from the cross ‘Zhongke 311’ × ‘Xinong 511’ was used as experimental material. The population had a relatively concentrated flowering period. The day on which the central spikelets of the main-stem spikes of ≥95% of the plants in the population flowered (anthers visible) was designated as the uniform flowering date (0 DAA). At 23 DAA, three relatively uniformly developed seeds from the middle portion of the spike were selected per line, placed in 24-well cell culture plates, and germinated according to the optimized protocol (drying at 40 °C for 2 d + moist pre-treatment at 4 °C for 2 d + soaking in 1% H_2_O_2_ for 1 d). Germination was assessed 2 d after the start of the germination test (pre-experiments indicated this time point was sufficient to evaluate germination status and uniformity, facilitating prompt sowing to avoid damage from excessive radicle and plumule elongation during transplanting). One well-germinated seed per line was selected and sown singly into a 200-cell plug tray. The seedling emergence rate was recorded after 10 d of culture under a 22 h light/2 h dark photoperiod at 22 °C.

### 2.4 Data analysis

Statistical analysis was performed using SPSS 26.0. Single-factor experiments (Section 2.3.1) and the multi-cultivar validation experiment (Section 2.3.4) were analyzed using one-way analysis of variance (One-way ANOVA), and multiple comparisons were performed using the Tukey HSD method. When Levene’s test indicated heterogeneity of variances (*P*< 0.05), Welch’s ANOVA was used for the overall test, and the Games-Howell method was applied for multiple comparisons. The optimal germination-promoting combination screening experiment (Section 2.3.3), involving multiple pairwise comparisons among several treatments, was analyzed using one-way ANOVA followed by the Bonferroni method for multiple comparisons. Orthogonal experimental data (Section 2.3.2) were analyzed using a combination of range analysis and three-way ANOVA, with each replicate observation serving as the analytical unit to test the main effects of each factor. The *F*-value, *P*-value, and partial *η*² of each effect were calculated, with partial *η*² ≥ 0.14 considered a large effect (Cohen, 1988). For factors with significant main effects, the Bonferroni method was used for *post-hoc* multiple comparisons. Because the L_16_(4³) orthogonal array is a main-effect screening design, it does not provide sufficient degrees of freedom to reliably estimate two-factor interactions without confounding; therefore, the ANOVA model included only the three main effects. In particular, the potential interaction between drying duration (Factor B) and moist pre-treatment duration (Factor C) was not tested. Consequently, the selected treatment combination should be interpreted as the best among the tested level combinations under the main-effects model, not as a statistically proven global optimum.

The significance level was uniformly set at *α* = 0.05; in the results, *P<* 0.01 indicates that the difference reached a highly significant level. Graphs were plotted using OriginPro 2018.

## 3 Results

### 3.1 Single-factor experiment results

#### 3.1.1 Effect of harvest time on wheat seed germination

Significant changes in spike and seed morphology were observed over time after anthesis. The period from 20 to 25 DAA was a critical stage during which spikes and seeds turned from green to yellow, marking the onset of the maturation phase (**Figures 1A, B**). Hundred-grain weight increased continuously with time after anthesis, from 3.08 g at 15 DAA to 4.62 g at 25 DAA, after which the rate of increase slowed, reaching a maximum of 4.82 g at 30 DAA. No significant differences were found among 25, 30, and 35 DAA (**Figure 1C**; **Supplementary Table S2**), indicating that dry matter accumulation was essentially complete by approximately 25 DAA.

**Figure 1:**
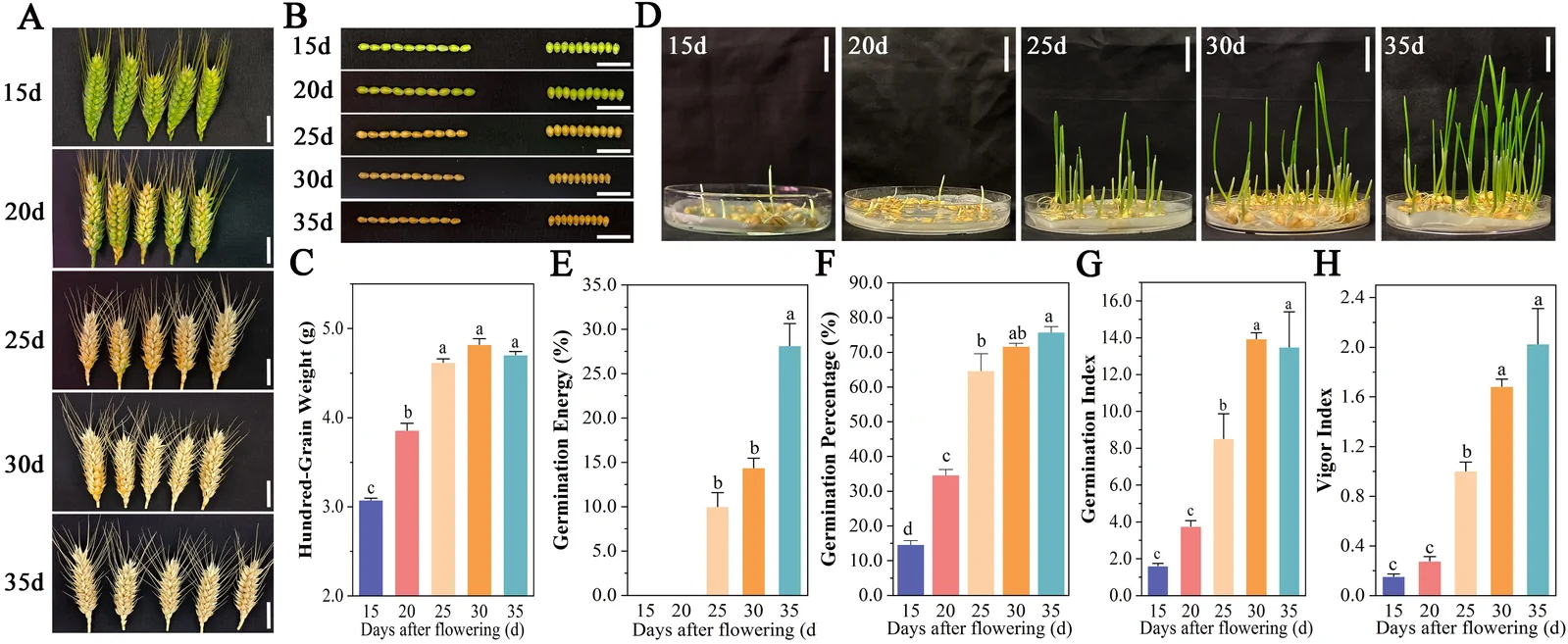
Effect of different harvest times on seed germination. **(A)** Fresh spikes harvested at 15, 20, 25, 30, and 35 DAA; **(B)** Fresh seeds harvested at the corresponding time points; **(C)** Hundred-grain weight of seeds at different harvest times; **(D)** Photographs of seed germination on day 7 of the standard germination test following spike drying at 40 °C for 5 d and threshing; **(E)** Germination energy of seeds at different harvest times; **(F)** Germination percentage of seeds at different harvest times; **(G)** Germination index of seeds at different harvest times; **(H)** Vigor index of seeds at different harvest times. Scale bars in **(A, B, D)** = 2 cm. In **(C, E, F, H)** different letters indicate highly significant differences among treatments (*P*< 0.01, Tukey HSD test). For **(G)** (germination index), heterogeneity of variances was detected; therefore, Welch’s ANOVA followed by the Games-Howell test was used, and different letters indicate significant differences at *P*< 0.05.

Analysis of seed germination indices showed that germination energy, germination percentage, germination index, and vigor index all increased with prolonged grain-filling duration (**Figures 1D-H**; **Supplementary Table S3**). The germination percentage of seeds harvested at 15 DAA was only 14.64%. The period 20–25 DAA represented a critical window for the enhancement of germination capacity: germination percentage increased sharply from 34.63% at 20 DAA to 64.67% at 25 DAA (*P*< 0.01), representing a relative increase of 86.7%. Thereafter, all germination indices continued to rise, reaching their highest values within the tested time range at 35 DAA (mean germination percentage 75.78%, germination energy 28.17%, germination index 13.49, vigor index 2.03).

These results indicate that germination capacity continued to improve until 35 DAA, even after hundred-grain weight had stabilized at 25 DAA. This suggests that under SB conditions, physiological maturity of wheat seeds (marked by maximum dry matter accumulation) and germination capacity maturity (marked by complete dormancy release) are two asynchronous processes; the latter requires a longer grain-filling duration on the mother plant. This finding provides an important basis for determining the optimal harvest node that balances generation cycle and seed vigor.

#### 3.1.2 Effect of drying temperature on wheat seed germination

To identify the optimal post-harvest drying temperature, the effects of air-drying at room temperature (22 ± 1 °C) and drying at 30, 35, 40, 45, 50, 55, and 60 °C for 3 d on seed germination were compared (seeds harvested at 30 DAA). The results showed that germination percentage, germination energy, germination index, and vigor index all exhibited a trend of initially increasing and then decreasing with increasing drying temperature, peaking at 40 °C (**Figures 2A–E**; **Supplementary Table S4**). The germination percentage (68.89%), germination energy (30.00%), germination index (6.53), and vigor index (1.22) under the 40 °C treatment were all significantly higher than those under all other temperature treatments (*P*< 0.01). The germination percentage of the room temperature air-drying treatment was 43.33%, which was not significantly different from the 30 °C and 35 °C treatments, but was significantly lower than that of the 40 °C treatment. When the temperature reached 45 °C or above, all indices dropped sharply, indicating that high-temperature (≥ 45 °C) drying severely damages seed germination capacity.

**Figure 2:**
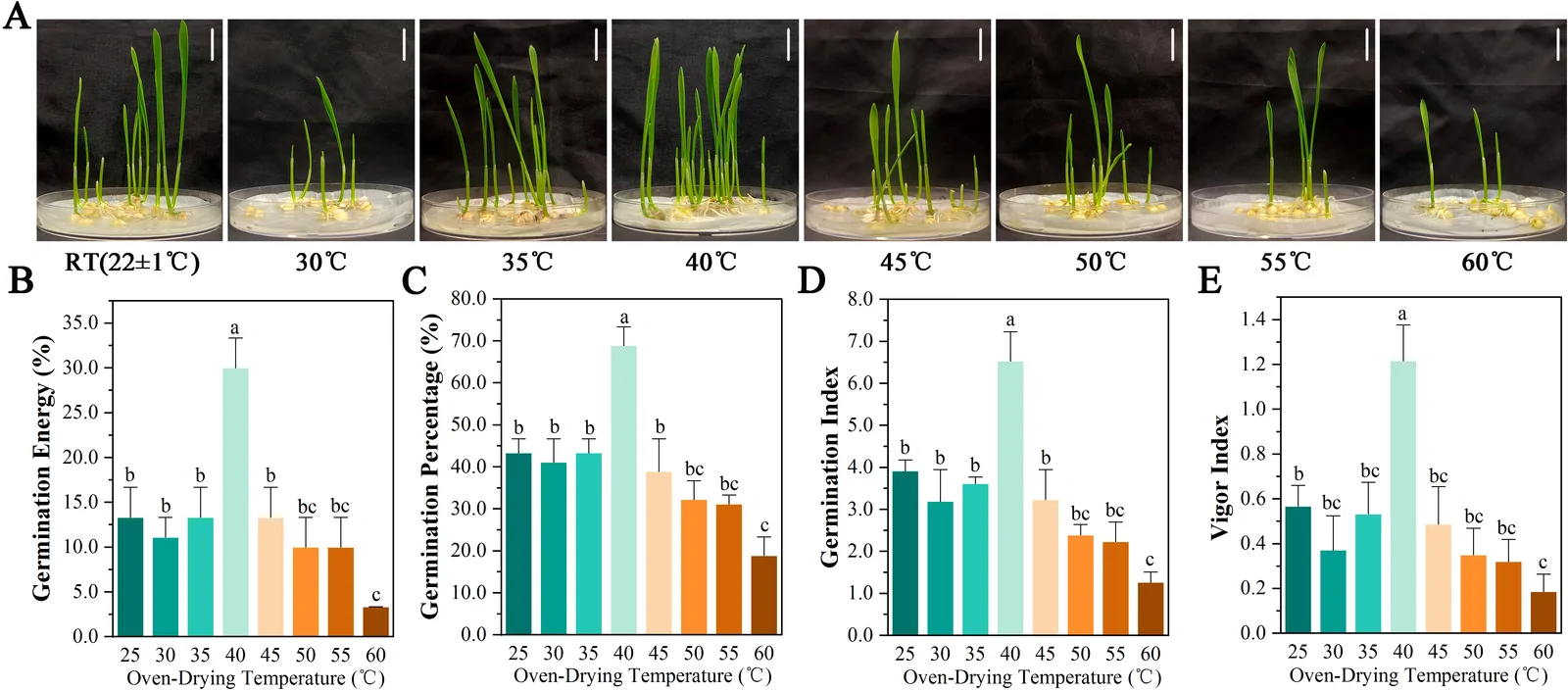
Effect of drying temperature on seed germination. **(A)** Photographs of seed germination on day 7 after different temperature treatments (room temperature air-drying and drying at 30-60 °C); **(B)** Germination energy of each treatment; **(C)** Germination percentage of each treatment; **(D)** Germination index of each treatment; **(E)** Vigor index of each treatment. Scale bar = 2 cm. Different letters indicate highly significant differences among treatments (*P*< 0.01, Tukey HSD test).

#### 3.1.3 Effect of drying duration at 40 °C on wheat seed germination

The dehydration process and germination response of seeds harvested at 30 DAA and dried at 40 °C are shown in **Figure 3**. Water loss was most rapid during the early stage of drying (0-0.5 d), with an average weight loss of 8.62%; thereafter, the dehydration rate slowed, and constant weight was essentially reached by 2.5 d (weight loss rate 10.51%, 11.43% after 6 d; **Figure 3A**; **Supplementary Table S5**).

**Figure 3:**
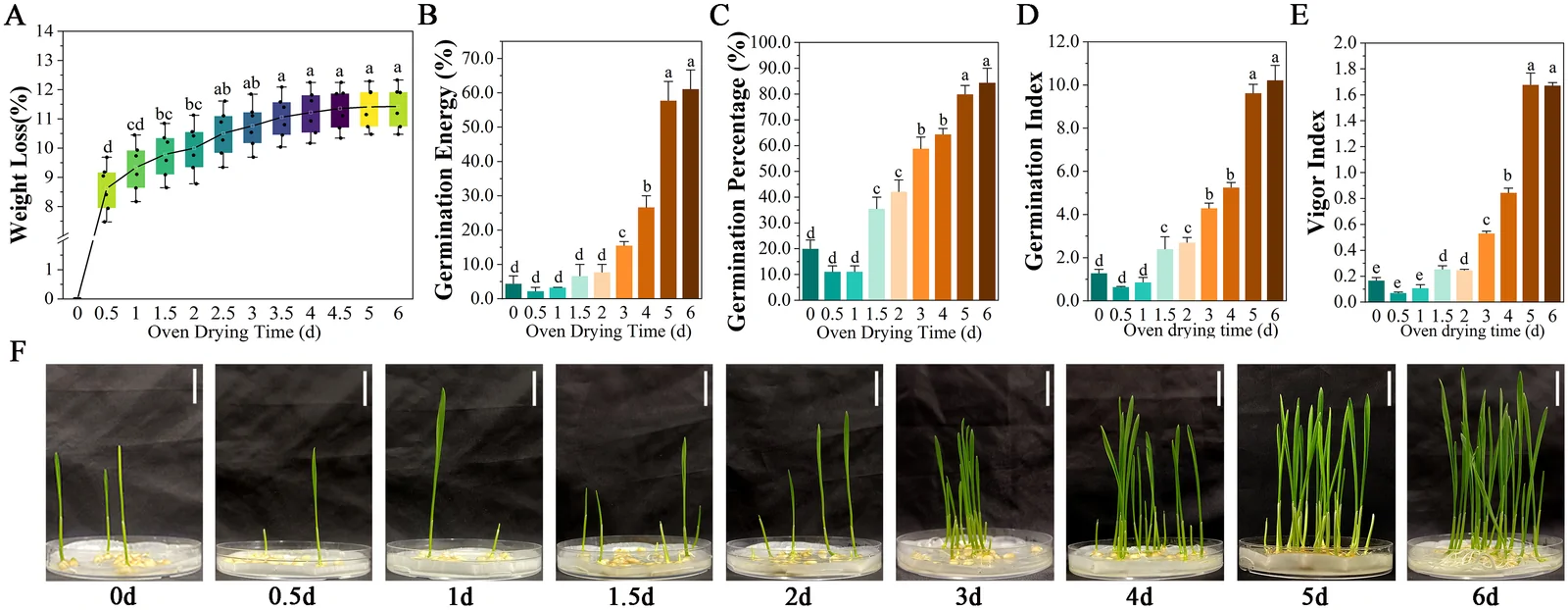
Effect of drying duration at 40 °C on seed germination. **(A)** Dynamic changes in relative seed weight (weight loss rate) during drying at 40 °C; **(B)** Germination energy at different drying durations; **(C)** Germination percentage at different drying durations; **(D)** Germination index at different drying durations; **(E)** Vigor index at different drying durations; **(F)** Photographs of each treatment on day 7 of germination (scale bar = 2 cm). For panels B and C, Levene’s test confirmed homogeneity of variances (P > 0.05); therefore, one-way ANOVA with Tukey HSD test was used, and different letters indicate highly significant differences among treatments (*P*< 0.01). For panels D and E, heterogeneity of variances was detected (Levene’s test, *P*< 0.05); therefore, Welch’s ANOVA followed by the Games-Howell test was applied, and different letters indicate highly significant differences among treatments (*P*< 0.01).

Germination test results showed that as drying time increased, germination energy and germination percentage decreased slightly in the early stage of drying (0–1 d) and then gradually increased (**Figures 3B, C**; **Supplementary Table S6**). Germination percentage reached 58.89% (exceeding 50%) at 3 d of drying, increased significantly to 80.00% at 5 d, and reached 84.44% at 6 d; no significant difference was observed between 5 d and 6 d (*P* > 0.05), indicating that drying for 5 d could already achieve a satisfactory germination effect. The germination percentages of the 5 d and 6 d drying treatments were both significantly superior to those of the other drying durations. The trends of germination index and vigor index were consistent with that of germination percentage.

Seed dehydration and the improvement of germination capacity were not entirely synchronous. Although seeds essentially reached constant weight by 3 d, germination percentage did not plateau at that point but continued to rise until 5 d, indicating that simply reaching constant weight through water loss is insufficient to completely release seed dormancy; an additional post-ripening period (approximately 1.5 d) after drying is required to fully unlock germination potential. Therefore, if maximum germination percentage is the goal, drying for 5 d is necessary; if time efficiency is also a consideration, drying for 3 d achieves a germination percentage exceeding 50% (58.89%), saving 2 d of processing time compared to 5 d, representing a practical approach that balances seed vigor and generation cycle in SB.

#### 3.1.4 Effect of moist pre-treatment duration at 4 °C on wheat seed germination

For seeds harvested at 30 DAA and subjected to moist pre-treatment at 4 °C, germination percentage, germination energy, germination index, and vigor index all increased progressively as the treatment duration increased from 0 to 4 d (**Figures 4A–E**; **Supplementary Table S7**). The germination percentage of the 4 d treatment (95.94%) was significantly higher than that of the 3 d treatment (83.25%, *P*< 0.01). The trends of germination index and vigor index were consistent with that of germination percentage (**Figures 4C, D**). Although the 4 d treatment yielded the best results, from the perspective of accelerating generation cycles, it is necessary to strike a balance between germination percentage and treatment duration, which will be further explored in the subsequent multi-factor optimization.

**Figure 4:**
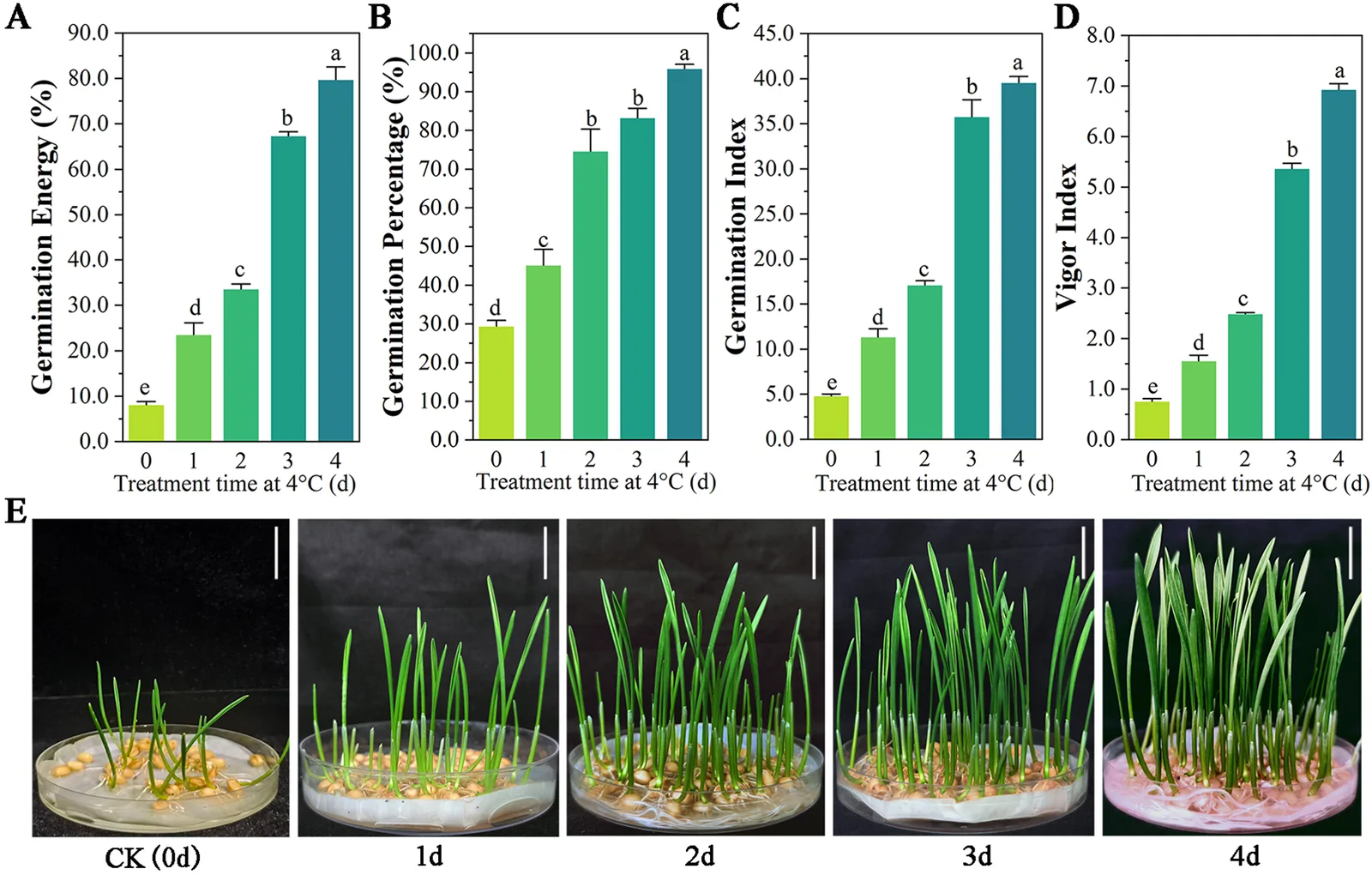
Effect of moist pre-treatment duration at 4 °C on seed germination. **(A)** Germination energy at different treatment durations; **(B)** Germination percentage at different treatment durations; **(C)** Germination index at different treatment durations; **(D)** Vigor index at different treatment durations; **(E)** Photographs of each treatment on day 7 of germination (scale bar = 2 cm). Different letters indicate significant differences (*P*< 0.01, Tukey HSD test).

### 3.2 Multi-factor orthogonal experiment optimization results

Based on the single-factor experiments that clarified the role of each factor, an L_16_(4³) orthogonal array was used to systematically investigate the combined effects and main effects of grain-filling duration after anthesis (A), drying duration at 40 °C (B), and moist pre-treatment duration at 4 °C (C) on seed germination (see **Table 1** for the design). The germination percentage (mean ± SD) of each treatment combination is detailed in **Supplementary Table S8**. Germination percentages ranged from 0% to 66.38%.

The results showed that for seeds harvested at 17 and 19 DAA, after corresponding treatments, only one treatment (Treatment 8, A_2_B_4_C_3_) achieved a germination percentage of 35.59%, while the remaining treatments showed either almost no germination or extremely low germination percentages (**Figure 5**). This indicates that seeds with these grain-filling durations remained in a state of deep dormancy even after germination-promoting treatments. For seeds harvested at 21 DAA, germination percentages varied widely among treatments, ranging from 1.67% to 47.39%, indicating that this harvest stage did not consistently produce high germination. Treatment 10 achieved 47.39% and did not differ significantly from the optimal treatment group harvested at 23 DAA (P > 0.05). In contrast, most treatments harvested at 23 DAA achieved higher germination percentages. Among them, Treatments 13, 14, and 15 achieved germination percentages of 62.05%, 66.37%, and 59.63%, respectively, with no significant differences among the three (*P* > 0.05), and all were significantly superior to Treatments 4, 8, 9, 11, and 12 (*P*< 0.05). Treatment 16 (47.31%) did not differ significantly from Treatment 10 (47.39%). Although these three treatments corresponded to different combinations of drying and moist pre-treatment durations, they were all based on harvest at 23 DAA, suggesting that once the key prerequisite of sufficient grain-filling duration is satisfied, drying and moist pre-treatments can be combined within a certain parameter range to achieve high germination levels. This also suggests that the effects of drying and moist pre-treatment are not purely additive, and potential interactions cannot be excluded. **Figure 5B** shows the germination of Treatments 1, 8, 10, and 14 on day 7 of the standard germination test: Treatment 1 showed no germination, Treatments 8 and 10 showed partial but non-uniform germination, while Treatment 14 exhibited visibly more consistent and high germination, visually confirming the decisive effect of grain-filling duration on seed germination. Given that multiple treatment combinations based on harvest at 23 DAA demonstrated high germination potential, the next phase would further screen for a practical protocol balancing high germination percentage and high time-efficiency on this basis, taking into account the length of the treatment cycle.

**Figure 5:**
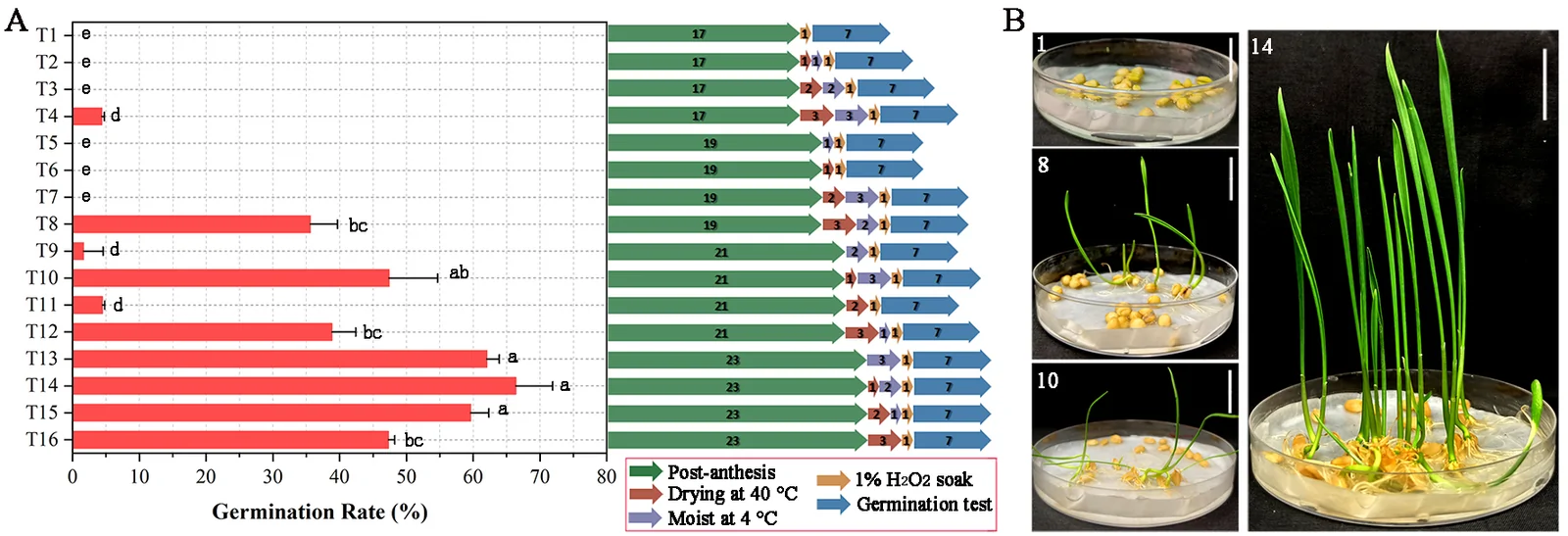
Germination performance of wheat under different grain-filling durations and treatments. **(A)** Germination percentage of each treatment and schematic diagram of grain-filling and experimental workflow (numbers on arrows indicate the duration of each stage in days). Levene’s test indicated heterogeneity of variances (*P*< 0.05); therefore, Welch’s ANOVA followed by the Games-Howell test was applied. Different letters indicate significant differences among treatments at the *P*< 0.05 level. **(B)** Photographs of seed germination on day 7 of the standard germination test for each treatment (scale bar = 2 cm).

Range analysis (**Table 3**) showed that the range value (*R*) of factor A (57.75%) was considerably larger than that of factor B (15.60%) and factor C (15.50%), reconfirming that harvest time is the most critical limiting factor. The optimal levels for each factor were A_4_ (23 d), B_4_ (3 d), and C_4_ (3 d), yielding a theoretically inferred combination A_4_B_4_C_4_, which was not directly tested in the subsequent validation experiment. However, both range and variance analyses confirmed the absolute dominance of factor A, while the effect sizes of factors B and C were relatively limited. This implies that, provided factor A is at its optimal level (A_4_), there is room for flexible optimization of the combinations of B and C based on efficiency or cost considerations. Range analysis was used only as a descriptive method to rank the relative importance of the three factors; formal statistical inference was based on the three-way ANOVA.

**Table 3:** Effects of different treatment factors on wheat seed germination percentage (based on range analysis of orthogonal experiment).

Factor	Level	Mean germination percentage (%)	Range (*R*)	Order of importance
A: Days after anthesis (d)	17	1.09	57.75	A > B ≈ C
	19	8.90		
	21	23.10		
	23	58.84		
B: Drying at 40 °C (d)	0	15.93	15.60	
	1	28.44		
	2	16.03		
	3	31.53		
C: Moist pre-treatment at 4 °C (d)	0	12.95	15.50	
	1	24.63		
	2	25.91		
	3	28.45		

Mean germination percentage for each factor level is based on 12 observations (4 treatment combinations × 3 biological replicates). The range (*R*) is the difference between the maximum and minimum mean germination percentages at each level for a given factor, reflecting the intensity of the factor’s effect.

Three-way analysis of variance (**Table 4**) further confirmed that factor A had a large effect on germination percentage (partial η² = 0.837; *F* = 65.17, *P*< 0.001), according to the conventional threshold of 0.14 for a large effect (Cohen, 1988). Although factors B and C also reached significant levels (*P*< 0.01), their effect sizes were much smaller than that of factor A (partial *η*² = 0.345 and 0.271, respectively). The coefficient of determination of the three-factor main effect model was *R*² = 0.858, indicating that, without considering interaction effects, the main effects of grain-filling duration, drying duration, and moist pre-treatment duration could explain 85.8% of the total variation in germination percentage, demonstrating good model fit. This confirms that analysis based on main effects can reliably identify the key influencing factors.

**Table 4:** Analysis of variance (ANOVA) for germination percentage.

Source	Sum of squares (SS)	df	Mean square (MS)	*F*-value	*P*-value	Partial *η*²
A: Days after anthesis	23561.883	3	7853.961	65.173	< 0.001	0.837
B: Drying duration	2412.386	3	804.129	6.673	0.001	0.345
C: Moist pre-treatment duration	1701.770	3	567.257	4.707	0.007	0.271
Error	4579.376	38	120.510			
Corrected Total	32255.415	47				
Model (*R*²)						0.858

The uncorrected total sum of squares was 57,612.927 (df = 48). The corrected total sum of squares (32,255.415, df = 47) equals the sum of the A, B, C, and error sums of squares. Partial *η*² is reported as an effect size measure for each factor; values ≥ 0.14 are considered large (Cohen, 1988). It does not represent the proportion of total variance explained. Model *R*² = 0.858, Adjusted *R*² = 0.824.

Bonferroni *post-hoc* multiple comparisons (**Table 3**) showed that the mean germination percentage of the A_4_ treatment (23 DAA; 58.84%) was significantly higher than that of A_1_ (17 DAA; 1.09%), A_2_ (19 DAA; 8.90%), and A_3_ (21 DAA; 23.10%) (*P*< 0.001), while the differences among A_1_, A_2_, and A_3_ were not significant (*P* > 0.05). This statistically confirms that delaying harvest to 23 DAA is a prerequisite for breaking seed dormancy and achieving high germination percentage; only on this basis can drying and moist pre-treatment exert further combined effects. The main effects of the three factors on germination percentage are shown in **Supplementary Figure S2**: as grain-filling duration increased from 17 to 23 d, germination percentage rose sharply and continuously; drying duration at 40 °C showed better effects at 1 and 3 d, with a slight decrease at 2 d; moist pre-treatment duration at 4 °C gradually improved germination percentage with prolonged treatment, but the increase was relatively gradual.

It should be noted that this orthogonal experiment was designed to reveal the main effects and optimal levels of each factor, with a maximum germination percentage of 66.37%. Because the L_16_(4³) design does not allow testing of interaction effects, the identified optimal levels should be interpreted within the main-effects framework. The reported optimal combination may depend on interactions that were not evaluated in this design. Based on the identified earliest effective harvest time (23 DAA) among the tested treatments, subsequent research would specifically refine the combinations of drying and moist pre-treatment to explore practical protocols that further enhance germination percentage and overall efficiency.

### 3.3 Validation and efficiency analysis of the optimized combination

Based on the earliest effective harvest time identified among the tested treatments (23 DAA), six combinations of drying and moist pre-treatment durations were validated, and the results are presented in **Table 5** (see also **Supplementary Table S9**). Germination percentages of all combinations exceeded 73%. Among them, Treatments 3 (A_4_B_3_C_3_, drying 2 d + moist pre-treatment 2 d), 4 (A_4_B_3_C_4_, drying 2 d + moist pre-treatment 3 d), 5 (A_4_B_4_C_3_, drying 3 d + moist pre-treatment 2 d), and 6 (A_4_B_4_C_4_, drying 3 d + moist pre-treatment 3 d) all achieved germination percentages above 97%, with no significant differences among the four (*P* > 0.05); however, all four were significantly higher than Treatment 1 (73.98%) and Treatment 2 (89.65%) (*P*< 0.05). Among these, Treatment 3 had the lowest coefficient of variation (*CV*, 0.35%), indicating the most stable performance.

**Table 5:** Germination performance of the six treatment combinations (based on harvest at 23 DAA).

Treatment	Combination	B: drying (d)	C: Moist pre-treatment (d)	Total duration T (d)¹	Germination (%) (Mean ± SD)	*CV* (%)	*EI*²
1	A_4_B_2_C_4_	1	3	35	73.98 ± 6.08 c	8.22	2.11
2	A_4_B_4_C_2_	3	1	35	89.65 ± 2.87 b	3.20	2.56
3	A_4_B_3_C_3_	2	2	35	97.06 ± 0.34 a	0.35	2.77
4	A_4_B_3_C_4_	2	3	36	97.53 ± 2.15 a	2.20	2.71
5	A_4_B_4_C_3_	3	2	36	97.38 ± 2.27 a	2.33	2.70
6	A_4_B_4_C_4_	3	3	37	98.81 ± 2.06 a	2.08	2.67

Mean ± SD are based on three biological replicates (n = 3). ¹Total duration T = Grain-filling 23 d + B + C + H_2_O_2_ treatment 1 d + Germination test 7 d = 31 + B + C; ²Efficiency Index *EI* = Germination percentage/Total duration T.

Different letters following data in the same column indicate significant differences at the *P*< 0.05 level based on the Bonferroni test.

In terms of the Efficiency Index (*EI*), Treatment 3 had the highest *EI* value (2.77), achieving a germination percentage of 97.06% within a total cycle of 35 days, representing optimal time-unit efficiency. Although Treatments 4, 5, and 6 had slightly higher germination percentages (97.53%-98.81%), their total cycles were extended to 36–37 days, and their *EI* values decreased to 2.60-2.71. Treatments 1 and 2, although sharing the same total duration of 35 days with Treatment 3, had lower germination percentages (73.98% and 89.65%, respectively), resulting in inferior overall efficiency compared to Treatment 3 (*EI* = 2.11 and 2.56, respectively). Clearly, Treatment 3 achieved high germination percentage and the lowest within-treatment variability (CV = 0.35%) among the tested combinations, demonstrating outstanding overall advantages. Among the six combinations tested, Treatment 3 was identified as the best-performing practical protocol for high-vigor seed production of winter wheat under SB conditions (harvest at 23 DAA + drying at 40 °C for 2 d + moist pre-treatment at 4 °C for 2 d). The theoretically inferred combination A_4_B_4_C_4_ was not tested and thus remains uncertain. However, this optimum should be considered within the tested parameter space and the main-effects framework, as interaction effects were not evaluated.

### 3.4 Multi-cultivar applicability validation of the optimized protocol

Two controls were established: 23d-CK (with the same total post-harvest treatment duration as the optimized protocol to exclude the effect of treatment time) and 27d-CK (with the same total duration from anthesis to germination initiation as the optimized protocol to compare post-harvest treatment with extended grain-filling). It should be noted that 27d-CK did not include a drying step; therefore, this comparison reflects the overall difference between two time-allocation strategies—early harvest followed by post-harvest treatment versus extended grain-filling without post-harvest treatment—rather than the isolated effect of either grain-filling duration or post-harvest drying alone. The optimized protocol 23d-T (harvest at 23 DAA → drying at 40 °C for 2 d → moist pre-treatment at 4 °C for 2 d → 1% H_2_O_2_ treatment for 1 d) was validated in ten winter wheat cultivars. The results showed that this protocol exhibited significantly higher germination energy and germination percentage than both controls across all tested cultivars (*P*< 0.01) (**Figure 6**; **Supplementary Table S10**). Under the optimized protocol, germination energy of the ten cultivars ranged from 58.08% to 89.54%, and germination percentage from 80.57% to 95.69%, with seven cultivars achieving germination percentages above 85%. In contrast, 23d-CK showed only 1.33%–23.96% germination energy and 2.62%–37.92% germination percentage; 27d-CK exhibited 3.20%–44.44% germination energy and 10.63%–57.56% germination percentage. Thus, although the optimized protocol outperformed both controls, the comparison with 27d-CK should be interpreted as a practical advantage of the combined early-harvest plus post-harvest treatment strategy over extended grain-filling without treatment, rather than as evidence that post-harvest treatment per se is superior to continued grain-filling. Taken together, the optimized post-harvest treatment protocol established in this study possesses broad applicability and good stability across different winter wheat genotypes under SB conditions.

**Figure 6:**
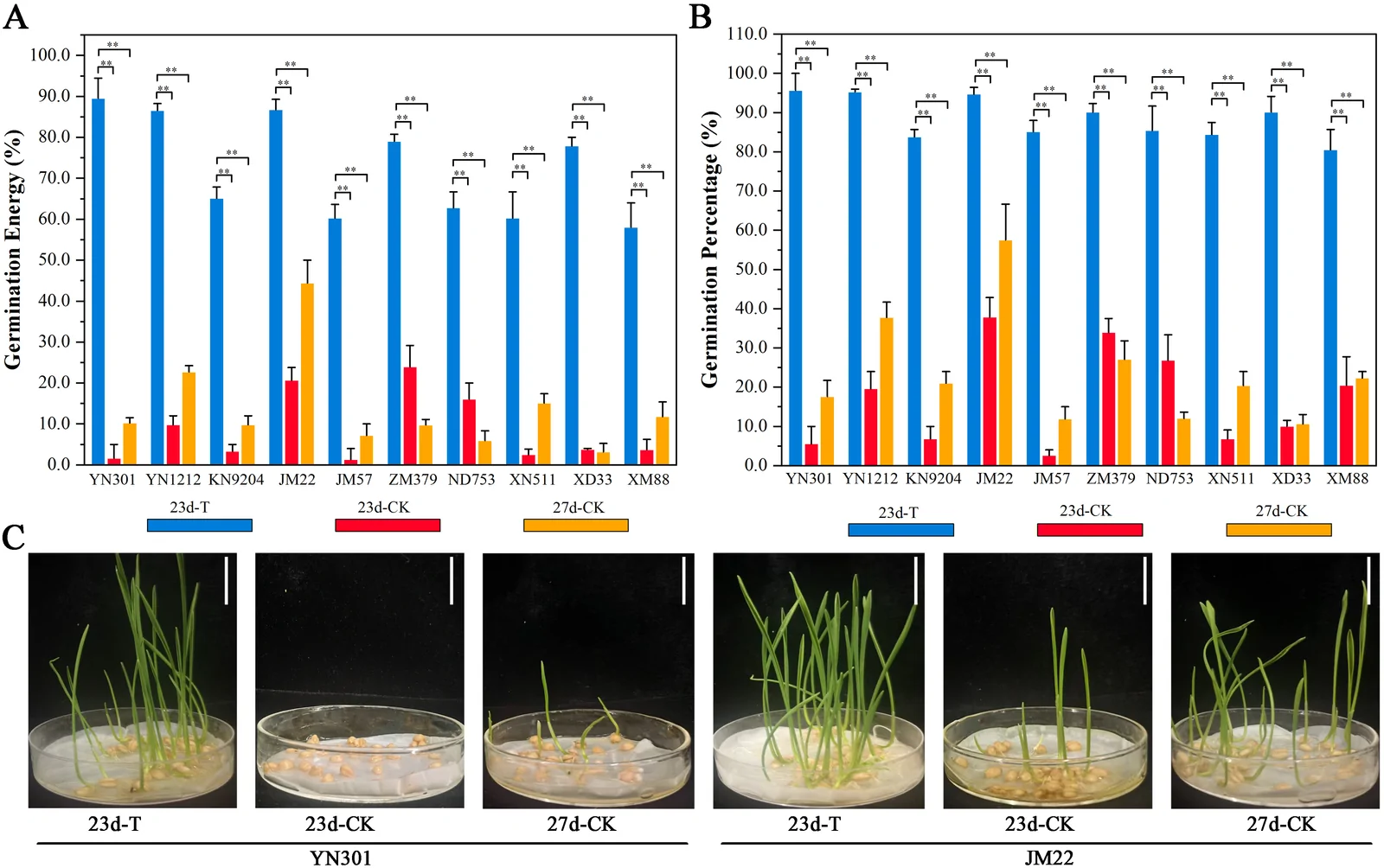
Germination performance of ten winter wheat cultivars under the optimized protocol. **(A)** Germination energy; **(B)** Germination percentage. In **(A, B)** ** indicates significant difference at the 0.01 level (*P*< 0.01, Tukey HSD test). **(C)** Representative photographs of YN301 and JM22 on day 7 of germination. Scale bar = 2 cm. In the figure, 23d-T represents the optimized protocol: harvest at 23 DAA, drying at 40 °C for 2 d, moist pre-treatment at 4 °C for 2 d, and 1% H_2_O_2_ treatment for 1 d; 23d-CK: harvest at 23 DAA, air-drying at room temperature for 4 d, and 1% H_2_O_2_ treatment for 1 d; 27d-CK: harvest at 27 DAA and 1% H_2_O_2_ treatment for 1 d.

### 3.5 Generation advancement validation of the optimized protocol in an SSD population

The pre-harvest field growth of the tested F_3_ recombinant inbred line population (derived from the cross ‘Zhongke 311’ × ‘Xinong 511’) is shown in **Figure 7A**. Among the 200 tested lines, all three seeds germinated normally in 180 lines (90.0%), two seeds germinated in 11 lines (5.5%), only one seed germinated in two lines (1.0%), and no seeds germinated in seven lines (3.5%). The germination status of representative seeds in 24-well cell culture plates is shown in **Figure 7B**. One germinated seed per line was selected for single-seed sowing; for the seven lines with no seed germination, all three ungerminated seeds were sown into the soil. After 10 days of culture, the overall growth of plug seedlings is shown in **Figure 7C**, with a line-based seedling emergence rate of 97.5% (195/200 lines; i.e., the percentage of lines with at least one emerged seedling). The 21 seeds from the seven lines that showed no germination in the standard germination test were also sown. Of these seven lines, two eventually produced seedlings after sowing, while the remaining five did not emerge. Since individual seeds were not tracked, a seed-based emergence rate cannot be accurately calculated from the current data. The seedlings grew uniformly and robustly, and no obvious weak or malformed seedlings were observed. These results indicate that the optimized protocol provides stable and efficient single-seed germination support for SSD populations, effectively reduces the risk of generation interruption caused by insufficient seed vigor, and supports line advancement under the tested conditions.

**Figure 7:**
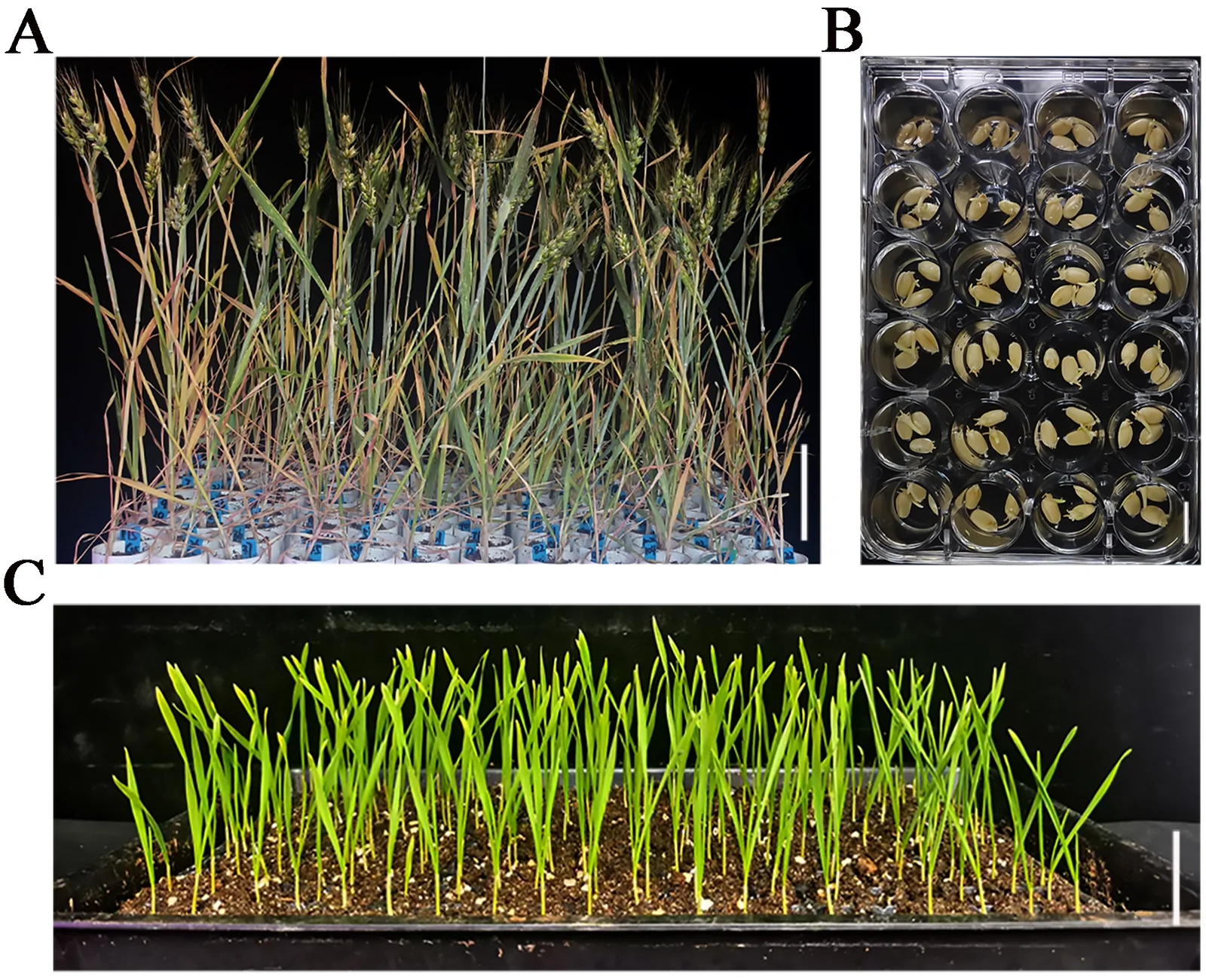
Validation of the optimized protocol for generation advancement in an SSD population. **(A)** Pre-harvest wheat plants, bar = 10 cm; **(B)** Germination status of representative seeds in 24-well cell culture plates, bar = 1 cm; **(C)** Overall growth of plug seedlings at 10 days after single-seed sowing, bar = 5 cm.

## 4 Discussion

### 4.1 Harvest time is the key factor determining seed germination capacity

Seed physiological maturation is a complex biological process involving multiple aspects such as storage compound accumulation, acquisition of desiccation tolerance, and the establishment and release of dormancy (Finch‐Savage and Leubner‐Metzger, 2006; Sripathy and Groot, 2023). This study found a significant asynchrony between physiological maturity and germination capacity maturity in winter wheat seeds under SB conditions. Although hundred-grain weight stabilized around 25 DAA, seed germination percentage continued to increase until 35 DAA. The period from 20 to 25 DAA was a critical window during which germination capacity surged from 34.63% to 64.67%. This indicates that under artificially accelerated growth conditions, the completion of dry matter accumulation is not equivalent to the complete release of dormancy and acquisition of germination capacity. The latter requires a longer grain-filling duration to complete physiological and biochemical processes such as embryo after-ripening, abscisic acid (ABA) degradation, and gibberellin (GA) synthesis; however, these mechanisms were not directly measured in this study.

Seed vigor does not peak at maximum dry matter accumulation but continues to improve during the subsequent maturation drying phase (Finch‐Savage and Leubner‐Metzger, 2006; Sripathy and Groot, 2023). Prematurely harvested seeds might fail to release physiological dormancy effectively, possibly because of higher ABA levels or insufficient GA synthesis as suggested in previous studies (Finch‐Savage and Leubner‐Metzger, 2006; Wang et al., 2018). This study further confirmed that grain-filling duration exerts a decisive influence on seed germination percentage: seeds harvested at 15 DAA had a low germination percentage (14.64%), whereas delaying harvest to beyond 25 DAA significantly increased the germination percentage to above 64.67%. In SB studies, the critical node for early harvest varies markedly among different crops. González-Barrios et al. (2020) found that oat (Avena sativa L.) harvested at 21 DAA could yield seeds sufficient for SSD (González‐Barrios et al., 2020). Gopinathan et al. (2025) further identified 21 DAA as the key node for obtaining sufficient surviving seeds for SSD in barley in the shortest time (Gopinathan et al., 2025). Watson et al. (2018) reported that ten spring wheat cultivars harvested prematurely at 14 DAA achieved germination rates of 80%-100% after cold stratification (Watson et al., 2018). In the present study, the winter wheat cultivar ‘Yannong 301’ harvested at 17 DAA and subjected to pre-treatments achieved only 35% germination, requiring a delay to 23 DAA to stably obtain high germination percentages. The substantial differences in germination percentages across studies may be attributed to genotypic differences. Notably, spring-habit and winter-habit cultivars may possess fundamental differences in ABA accumulation and GA synthesis kinetics during early grain-filling, warranting further in-depth investigation. These findings confirmed that grain-filling duration is the dominant factor controlling germination capacity in early-harvested winter wheat under SB.

It is noteworthy that while this study established the critical role of grain-filling duration under controlled conditions, seed germination under SB is also influenced in practice by the interactive effects of cultivation factors such as light, temperature, substrate moisture, and nutrient status. This partly explains why germination percentages are not entirely consistent across different planting batches, even when the same germination treatment is applied. Therefore, 23 DAA should be regarded as the earliest harvest node tested in our orthogonal design that achieved high germination when combined with post-harvest treatments. This node was not directly included in the single-factor harvest time experiment. If the breeding program demands a high generation advancement rate, this node can guarantee an acceptable germination percentage within the shortest cycle; if a higher germination percentage is required and the cycle length is less constrained, the grain-filling duration can be appropriately extended, for example to 25–30 DAA, to achieve greater germination assurance. These results were obtained under controlled SB conditions (22 °C, 22 h photoperiod, 60-70% RH, PPFD 250 μmol·m^-^²·s^-^¹), which should be considered when extrapolating to other environments.

### 4.2 Combined effects of drying and moist pre-treatment

Seed after-ripening is a key process for ensuring germination vigor, in which both drying and moist pre-treatment serve as widely recognized effective promoters (Buitink and Leprince, 2008; Ali et al., 2022). High-temperature drying can initiate after-ripening by reducing seed moisture content, promoting ABA degradation, and facilitating reactive oxygen species (ROS) signaling (Angelovici et al., 2010; Du et al., 2015; Ma et al., 2017; Bailly, 2019), whereas moist pre-treatment at low temperatures simulates natural stratification, thereby further alleviating residual physiological dormancy (Watson et al., 2018; González‐Barrios et al., 2020; Chen et al., 2022a; Gopinathan et al., 2025). On this basis, the present study is the first to quantify the best tested combination effects of these two treatments in a winter wheat SB system through an orthogonal experiment: 40 °C was the optimal drying temperature, and under the prerequisite of harvest at 23 DAA, the combination of drying at 40 °C for 2 d followed by moist pre-treatment at 4 °C for 2 d exhibited the best combined effect among those tested. Extending the treatment duration (3 d + 3 d) only marginally increased the germination percentage from 97.06% to 98.81%, a non-significant increase, but instead reduced overall efficiency due to the extended cycle length. The above post-harvest treatment workflow of “drying first, then moist pre-treatment at low temperature” effectively improved the germination percentage of early-harvested seeds and promoted a more consistent after-ripening process. Across replicates, the optimized protocol showed low within-treatment variability (CV = 0.35%; **Table 5**), which is consistent with a more consistent germination response, although this CV reflects variability among replicates rather than a direct measure of germination synchrony. This is particularly important for SB systems that adopt SSD as their core strategy, because consistent germination facilitates subsequent seedling management and reliable line advancement.

Although the main-effects model explained 85.8% of the variance in germination percentage, the possibility of an interaction between drying duration and moist pre-treatment duration cannot be ruled out. Biologically, the degree of seed dehydration may affect subsequent water uptake, membrane repair, and metabolic recovery during moist pre-treatment. In the present study, the comparable range values of Factor B and Factor C (15.60% vs 15.50%) and the lack of a significant difference between B_2_C_2_ (97.06%) and B_3_C_3_ (98.81%) collectively suggest that once a threshold of dehydration and moist pre-treatment is reached, further extension of either treatment provides only marginal benefits. Therefore, the protocol identified here should be regarded as a practical optimum within the tested parameter space and the main-effects framework, rather than a mathematically or biologically proven global optimum. Consequently, the reported optimal combination should be regarded as provisional, as its performance may depend on interactions that were not tested. Full factorial or response surface experiments would be required to more precisely characterize any potential interaction effects.

### 4.3 Application value and efficiency advantages of the optimized protocol

The efficient germination-promoting protocol finalized in this study is: “Harvest spikes at 23 DAA, dry at 40 °C for 2 d, thresh, apply moist pre-treatment at 4 °C for 2 d, and then soak in 1% H_2_O_2_ solution for 1 d.” During protocol screening, this study innovatively introduced the Efficiency Index (EI = germination percentage/total duration) as a comprehensive evaluation metric. Unlike conventional vigor indices that focus solely on final germination performance, EI incorporates grain-filling duration and post-harvest treatment duration into the evaluation system, better aligning with the core SB objective of “trading speed for generations” (Samantara et al., 2022).

The validation experiment confirmed the necessity of this evaluation strategy from multiple dimensions. First, although the theoretically optimal combination derived from range analysis (A_4_B_4_C_4_: harvest at 23 DAA + drying at 40 °C for 3 d + moist pre-treatment at 4 °C for 3 d) achieved the highest germination percentage (98.81%), its total duration reached 37 days, with an EI value of only 2.67, markedly lower than that of the optimal protocol (*EI* = 2.77). This demonstrates that a screening strategy based solely on germination percentage cannot identify the most time-efficient treatment combination in practice, further confirming the necessity of incorporating time cost into treatment protocol screening. Second, the A_4_B_3_C_3_ combination (i.e., the final optimized protocol) achieved a germination percentage of 97.06% within a total cycle of only 35 days, significantly exceeding the minimum germination standards required for SSD (Watson et al., 2018; González‐Barrios et al., 2020; Gopinathan et al., 2025). As noted in the Results, the 27d-CK comparison reflects the combined benefit of early harvest plus post-harvest treatment rather than the isolated effect of post-harvest treatment alone. This finding still supports the practical value of the optimized protocol, as it demonstrates that under the same total duration, allocating 4 d to post-harvest treatment after early harvest yields higher germination than allocating the same 4 d to extended grain-filling without any post-harvest treatment-a relevant comparison for SB time-allocation decisions. A more rigorous control design would further clarify the relative contributions of grain-filling duration and post-harvest drying. In terms of cultivar applicability, some variation was observed in the response of different cultivars to the optimized protocol. For example, the germination percentages of YN301, YN1212, and JM22 were significantly higher than those of JM57 and ND753, which may be related to genetic differences in seed maturation rate and dormancy characteristics under SB conditions. Nevertheless, germination percentages ranged from 80.57% to 95.69%, with seven of ten cultivars exceeding 85%, indicating the broad applicability of this protocol to different winter wheat genotypes.

In summary, this protocol not only meets the requirements for seed quantity and consistent germination in large-scale breeding population construction, but also maximizes generation advancement efficiency with the shortest treatment cycle, thereby expanding the application potential of SB technology in multi-generation cycling and precise phenotypic identification. Furthermore, validation in an SSD population confirmed that this protocol achieved a line-based seedling emergence rate of 97.5% across 200 segregating lines, supporting its applicability for single-seed descent advancement under the tested conditions. It should be noted that this validation was performed in a single F_3_ generation; further studies are needed to confirm performance over multiple successive cycles. In addition, the flowering time of the population used in this experiment was relatively concentrated, which facilitated uniform harvesting and batch processing. If the flowering time of the population varies widely, it is recommended to mark and record different flowering batches and formulate a batch harvest plan accordingly, to ensure that seeds from each batch are harvested at an appropriate grain-filling duration, thereby maximizing the overall population germination percentage and generation advancement efficiency. Importantly, the EI-based optimization trades a marginal reduction in germination percentage for a shorter cycle. For germplasm preservation or the creation of very rare genetic materials where every seed is critical, the harvest time should be extended to 25–30 DAA, and the treatment protocol of “3 days of drying + 3 days of moist pre-treatment at 4 °C” should be prioritized to achieve the absolute highest germination percentage.

The above results indicate that the efficiency index-oriented optimization strategy can jointly improve seed vigor and time efficiency in SB systems, providing an operational technical paradigm for efficient multi-generation cycling, as well as a methodological reference for addressing similar issues in SB systems of other crops.

## 5 Conclusion

Through systematic single-factor and multi-factor optimization experiments, this study identified grain-filling duration as the key factor limiting seed germination of winter wheat under SB, and established a practical post-harvest treatment workflow that enables high germination of early-harvested seeds. The optimized protocol substantially shortens the time from anthesis to germination test completion while achieving stable and high germination across multiple winter wheat cultivars. Validation in an SSD population further confirmed its reliability for rapid generation advancement. This workflow addresses the technical bottleneck of low germination in early-harvested seeds and provides a scalable seed production pipeline for large-scale population construction and efficient multi-generation cycling in SB.

## Data Availability

The original contributions presented in the study are included in the article/**Supplementary Material**. Further inquiries can be directed to the corresponding authors.
