# Supplementary material for: Optimization of post-ripening treatments for efficient germination of early-harvested winter wheat seeds under speed breeding
Source: Front Plant Sci. 2026 Sep 14;17:1940423. doi: 10.3389/fpls.2026.1940423 (PMC13617137; doi:10.3389/fpls.2026.1940423)
Supplement: Supplementary file 1 [file DataSheet1.docx]

Supplementary Fig. S1. Germination percentage of winter wheat seeds after different chemical pre-germination treatments.

Seeds of cultivar ‘Yannong 301’ were harvested at 30 days after anthesis, dried at 40°C for 3 d, then soaked in the indicated solutions for 24 h at room temperature (22 ± 1°C). Treatments included a water control (CK), gibberellic acid (GA₃) at 30–60 mg/L, and hydrogen peroxide (H₂O₂) at 0.5%–3%. Germination percentage was recorded after 7 d under standard germination conditions. Values are means ± SD (n = 3). Different letters above bars indicate significant differences among treatments (P < 0.05, Tukey HSD test).

Supplementary Fig. S2 Main effects of grain‑filling DAA (A), drying duration at 40 °C (B), and moist pre‑treatment duration at 4 °C (C) on wheat seed germination percentage.

Data points represent the mean germination percentage of each factor at the indicated levels, averaged across all other factor levels in the L₁₆(4³) orthogonal experiment (see Supplementary Table S8 for raw germination data). Error bars indicate standard deviation (SD, 𝑛=12 for each level). Different lowercase letters above the points within each panel indicate significant differences among levels (Games‑Howell post‑hoc test, P<0.05P<0.05). For factor A, level A₄ (23 DAA) resulted in a significantly higher germination percentage compared with A₁–A₃ (𝑃<0.001). Factors B and C also showed significant but much smaller effects.
