# Supplementary material for: Optimization of post-ripening treatments for efficient germination of early-harvested winter wheat seeds under speed breeding
Source: Front Plant Sci. 2026 Sep 14;17:1940423. doi: 10.3389/fpls.2026.1940423 (PMC13617137; doi:10.3389/fpls.2026.1940423)
Supplement: Supplementary file 2 [file Table1.docx]

**Supplementary Table S1 Information of the winter wheat cultivars used in this study**

| **Cultivar/Germplasm Name** | **Abbreviation** | **Origin/Breeder** | **Growth habit** | **Main characteristics** |
| --- | --- | --- | --- | --- |
| Yannong 301 | YN301 | Yantai Academy of Agricultural Sciences | Winter | High-yielding, lodging-resistant, hard grain, medium gluten (control) |
| Yannong 1212 | YN1212 | Yantai Academy of Agricultural Sciences | Semi-winter | Good lodging and cold tolerance, strong tillering, stable yield |
| Kenong 9204 | KN9204 | Institute of Genetics and Developmental Biology, CAS | Semi-winter | Compact plant type, good lodging resistance, high ear-bearing rate, immune to stem rust |
| Jimai 22 | JM22 | Shandong Academy of Agricultural Sciences | Semi-winter | Immune to powdery mildew, high tillering, high spike number, good grain appearance, stable yield |
| Jimai 57 | JM57 | Shandong Academy of Agricultural Sciences | Semi-winter | Medium-strong gluten, Fusarium head blight (FHB) resistant |
| Zhengmai 379 | ZM379 | Henan Academy of Agricultural Sciences | Semi-winter | Large grain, medium-long stability time, slow rust resistance, good lodging resistance |
| Nongda 753 | ND753 | China Agricultural University | Semi-winter | Strong gluten, early maturity, compact plant type, good grain fill, stable yield |
| Xinong 511 | XN511 | Northwest A&F University | Semi-winter | Strong gluten, moderate resistance to stripe rust, good lodging resistance, good ripening appearance, stable yield increase, high kernel number per spike |
| Xindong 33 | XD33 | Xinjiang Academy of Agricultural Sciences | Winter | Salt-alkali tolerant, lodging resistant, cold hardy, wide adaptability |
| Xinmai 88 | XM88 | Xinxiang Academy of Agricultural Sciences, Henan | Semi-winter | Strong gluten, dwarf, lodging and pre-harvest sprouting resistant, good disease resistance |

**Supplementary Table S2. Hundred‑grain weight (g) of winter wheat seeds harvested at different days after anthesis (DAA).**

Values are presented as mean ± standard deviation (SD), n = 3 biological replicates per harvest time. Each replicate consisted of five main‑stem spikes.

| Days after anthesis (DAA, d) | Mean 100-grain weight (g) ± SD (g) |
| --- | --- |
| 15 | 3.076 ± 0.036 |
| 20 | 3.859 ± 0.071 |
| 25 | 4.619 ± 0.043 |
| 30 | 4.823 ± 0.093 |
| 35 | 4.706 ± 0.032 |

**Note:** Grain number and grain weight per replicate were the sums of five main‑stem spikes. Hundred‑grain weight was calculated as (grain weight per replicate / grain number per replicate) × 100. Raw data for individual replicates are available in Supplementary Data (Excel file, Sheet “Exp1_HarvestTime_100GW”).

**Supplementary Table S3. Germination parameters of winter wheat seeds harvested at different days after anthesis (DAA).**

Values are presented as mean ± SD (n = 3). Germination rate, germination energy, germination index, and vigor index were calculated from raw germination counts and fresh weight.

| Harvest DAA | Germination energy (%) | Germination rate (%) | Germination index | Vigor index |
| --- | --- | --- | --- | --- |
| 15 | 0.00 ± 0.00 | 14.64 ± 1.08 | 1.63 ± 0.11 | 0.15 ± 0.02 |
| 20 | 0.00 ± 0.00 | 34.63 ± 1.47 | 3.77 ± 0.30 | 0.28 ± 0.03 |
| 25 | 10.02 ± 1.49 | 64.67 ± 4.52 | 8.53 ± 1.19 | 1.00 ± 0.10 |
| 30 | 14.40 ± 1.11 | 72.54 ± 2.31 | 13.94 ± 0.28 | 1.69 ± 0.06 |
| 35 | 28.17 ± 3.85 | 75.78 ± 1.47 | 13.49 ± 2.01 | 2.03 ± 0.27 |

**Footnote:** Raw germination data (daily counts, total seeds, fresh weight) are available in Supplementary Data (Excel file, Sheet “Exp2_HarvestTime_Germination_Raw”).

**Supplementary Table S4. Effect of drying temperature on germination parameters of winter wheat seeds.**

**Seeds were harvested at 30 days after anthesis and dried at different temperatures for 3 days. Room temperature (22°C) shade‑dried seeds served as the control. Values are presented as mean ± SD (n = 3).**

| Drying temperature (°C) | Germination energy (%) | Germination rate (%) | Germination index | Vigor index |
| --- | --- | --- | --- | --- |
| RT (CK) | 43.33 ± 5.77 | 13.33 ± 3.33 | 3.91 ± 0.31 | 0.57 ± 0.08 |
| 30 | 41.11 ± 5.09 | 11.11 ± 1.92 | 3.19 ± 0.66 | 0.37 ± 0.13 |
| 35 | 43.33 ± 3.33 | 13.33 ± 3.33 | 3.61 ± 0.17 | 0.53 ± 0.17 |
| 40 | 68.89 ± 5.09 | 30.00 ± 3.33 | 6.53 ± 0.87 | 1.22 ± 0.19 |
| 45 | 38.89 ± 6.94 | 13.33 ± 3.33 | 3.23 ± 0.64 | 0.49 ± 0.15 |
| 50 | 32.22 ± 5.09 | 10.00 ± 3.33 | 2.39 ± 0.35 | 0.35 ± 0.12 |
| 55 | 31.11 ± 3.85 | 10.00 ± 3.33 | 2.23 ± 0.43 | 0.32 ± 0.09 |
| 60 | 18.89 ± 5.09 | 3.33 ± 0.00 | 1.26 ± 0.29 | 0.19 ± 0.08 |

**Note:** Germination rate, germination energy, germination index, and vigor index were calculated from raw daily germination counts and fresh weight of seedlings. Raw data for individual replicates are available in Supplementary Data (Excel file, Sheet “Exp3_DryingTemp_Germination_Raw”).

**Supplementary Table S5. Cumulative water loss (%) of wheat seeds (50 seeds per sample) during drying at 40°C.**

Values are presented as mean ± SD (n = 6 independent samples).

| Drying time (h) | Cumulative water loss (%) |
| --- | --- |
| 0 | 0.00 ± 0.00 |
| 12 | 8.62 ± 0.83 |
| 24 | 9.34 ± 0.85 |
| 36 | 9.79 ± 0.83 |
| 48 | 10.00 ± 0.87 |
| 60 | 10.51 ± 0.85 |
| 72 | 10.76 ± 0.79 |
| 84 | 11.06 ± 0.77 |
| 96 | 11.22 ± 0.81 |
| 108 | 11.36 ± 0.76 |
| 120 | 11.41 ± 0.73 |
| 144 | 11.43 ± 0.74 |

**Note:** Cumulative water loss was calculated as (*W*0−*Wt*)/*W*0×100%, where *W*0 is the initial weight at 0 h and *Wt* is the weight at time *t*. Raw seed weight data for individual samples are available in Supplementary Data (Excel file, Sheet “Exp4_DryingDuration_WeightLoss_Raw”).

**Supplementary Table S6. Effect of drying duration at 40°C on germination parameters of winter wheat seeds.**

Seeds were harvested at 30 days after anthesis. Values are presented as mean ± SD (n = 3).

| Drying time (d) | Germination energy (%) | Germination rate (%) | Germination index | Vigor index |
| --- | --- | --- | --- | --- |
| 0 | 4.44 ± 1.92 | 20.00 ± 3.33 | 1.29 ± 0.20 | 0.17 ± 0.03 |
| 0.5 | 2.22 ± 1.92 | 11.11 ± 1.92 | 0.62 ± 0.01 | 0.07 ± 0.01 |
| 1 | 3.33 ± 0.00 | 11.11 ± 1.92 | 0.86 ± 0.20 | 0.11 ± 0.03 |
| 1.5 | 6.67 ± 3.33 | 35.56 ± 5.09 | 2.41 ± 0.50 | 0.25 ± 0.03 |
| 2 | 7.78 ± 1.92 | 42.22 ± 5.09 | 2.72 ± 0.21 | 0.25 ± 0.01 |
| 3 | 15.56 ± 1.92 | 58.89 ± 3.85 | 4.29 ± 0.20 | 0.53 ± 0.01 |
| 4 | 26.67 ± 3.33 | 64.44 ± 1.92 | 5.26 ± 0.24 | 0.85 ± 0.05 |
| 5 | 57.78 ± 5.09 | 80.00 ± 3.33 | 9.63 ± 0.66 | 1.68 ± 0.15 |
| 6 | 61.11 ± 5.09 | 84.44 ± 5.09 | 10.23 ± 0.64 | 1.67 ± 0.03 |

**Note:** Germination energy (GE) is the percentage of seeds germinated within the first 3 days; germination percentage (GP) is the percentage within 7 days. Raw germination data (daily counts and fresh weight) are available in Supplementary Data (Excel file, Sheet “Exp5_DryingTime_Germination_Raw”).

**Supplementary Table S7. Effect of low temperature (4 °C) treatment duration on germination parameters of winter wheat seeds.**

Seeds were harvested at 30 days after anthesis and treated at 4 °C for different durations, then germinated under the same conditions as in previous experiments. Values are presented as mean ± SD (n = 3). Germination energy (GE, %), germination rate (GR, %), germination index (GI), and vigor index (VI) were calculated from daily germination counts and fresh weight.

| Drying time (d) | Germination energy (%) | Germination rate (%) | Germination index | Vigor index |
| --- | --- | --- | --- | --- |
| 0 | 8.08 ± 1.07 | 29.43 ± 1.81 | 4.84 ± 0.29 | 0.76 ±0.07 |
| 1 | 23.58 ± 2.30 | 45.19 ± 3.51 | 11.34 ± 0.96 | 1.56 ± 0.15 |
| 2 | 33.64 ± 0.97 | 74.62 ± 5.87 | 17.12 ± 0.77 | 2.49 ± 0.02 |
| 3 | 67.36 ± 0.81 | 83.25 ± 2.14 | 35.79 ± 1.80 | 5.37 ± 0.12 |
| 4 | 79.74 ± 2.65 | 95.94 ± 1.74 | 39.56 ± 0.77 | 6.93 ± 0.11 |

Note: Germination energy (GE) = percentage of seeds germinated within the first 3 days. Germination rate (GR) = percentage of seeds germinated within 7 days. Germination index (GI) = Σ (Gt / Dt), where Gt is the number of germinated seeds on day t, Dt is the corresponding day. Vigor index (VI) = GI × average fresh weight per seedling (total fresh weight / total germinated seeds). Raw data (daily counts, total seeds, fresh weight) are available in Supplementary Data (Excel file, Sheet "Exp6_ColdTreatment_ Raw").

**Supplementary Table S8. Germination rate of winter wheat seeds under orthogonal design L₁₆(4³) with factors of harvest time after anthesis (A, d), drying duration at 40 °C (B, d), and moist pre‑treatment at 4 °C.**

Seeds were harvested at different days after anthesis (17, 19, 21, 23 d), then dried at 40 °C for 0–3 d, followed by cold (4 °C) treatment for 0–3 d. Germination rate (%) was recorded after 7 days. Values are presented as mean ± SD (n = 3).

| **Treatment No.** | **A (harvest DAA, d)** | **B (drying at 40 °C, d)** | **C (cold at 4 °C, d)** | **Germination rate (%)** |
| --- | --- | --- | --- | --- |
| 1 | 17 | 0 | 0 | 0.00 ± 0.00 |
| 2 | 17 | 1 | 1 | 0.00 ± 0.00 |
| 3 | 17 | 2 | 2 | 0.00 ± 0.00 |
| 4 | 17 | 3 | 3 | 4.37 ± 0.38 |
| 5 | 19 | 0 | 1 | 0.00 ± 0.00 |
| 6 | 19 | 1 | 0 | 0.00 ± 0.00 |
| 7 | 19 | 2 | 3 | 0.00 ± 0.00 |
| 8 | 19 | 3 | 2 | 35.59 ± 4.06 |
| 9 | 21 | 0 | 2 | 1.67 ± 2.89 |
| 10 | 21 | 1 | 3 | 47.39 ± 7.28 |
| 11 | 21 | 2 | 0 | 4.49 ± 0.30 |
| 12 | 21 | 3 | 1 | 38.87 ± 3.54 |
| 13 | 23 | 0 | 3 | 62.05 ± 1.86 |
| 14 | 23 | 1 | 2 | 66.38 ± 5.52 |
| 15 | 23 | 2 | 1 | 59.63 ± 2.70 |
| 16 | 23 | 3 | 0 | 47.31 ± 0.93 |

**Note:** Germination rate = (number of germinated seeds within 7 days / total seeds) × 100%. Raw data for each replicate (total seeds, 7‑day germinated seeds) are available in Supplementary Data (Excel file, Sheet "Exp7_Orthogonal_Raw").

**Supplementary Table S9. Validation of selected optimal treatment combinations based on harvest at 23 days after anthesis (DAA).**

Seeds were dried at 40 °C for different durations and then treated at 4 °C for different durations, as indicated. Germination rate (%) was recorded after 7 days. Values are presented as mean ± SD (n = 3). Different letters within the column indicate significant differences (P < 0.05, Tukey’s HSD). CV: coefficient of variation.

| **Treatment No.** | **B (drying at 40 °C, d)** | **C (cold at 4 °C, d)** | **Germination rate (%)** | **CV (%)** |
| --- | --- | --- | --- | --- |
| 1 | 1 | 3 | 73.98 ± 6.07 b | 8.20 |
| 2 | 3 | 1 | 89.65 ± 2.87 b | 3.20 |
| 3 | 2 | 2 | 97.06 ± 0.34 a | 0.35 |
| 4 | 2 | 3 | 97.53 ± 2.15 a | 2.20 |
| 5 | 3 | 2 | 97.38 ± 2.27 a | 2.33 |
| 6 | 3 | 3 | 98.81 ± 2.06 a | 2.08 |

Note: Treatment 1: A₄B₁C₃; Treatment 2: A₄B₃C₁; Treatment 3: A₄B₂C₂; Treatment 4: A₄B₂C₃; Treatment 5: A₄B₃C₂; Treatment 6: A₄B₃C₃ (A₄ = 23 DAA).

Means sharing the same letter are not significantly different (P > 0.05).

Raw data (total seeds and 7‑day germinated seeds per replicate) are available in Supplementary Data (Excel file, Sheet "Exp8_Validation_Raw").

**Supplementary Table S10. Germination performance of 10 winter wheat varieties under optimized combination (23d-T) and control treatments (23d-CK, 27d-CK).**

Seeds were harvested at 23 or 27 days after anthesis as indicated. The optimized treatment (23d-T) consisted of drying at 40 °C for 2 d followed by cold treatment at 4 °C for 2 d. Values are presented as mean ± SD (n = 3). Germination energy (GE, %) = percentage of seeds germinated within 3 days; germination rate (GR, %) = percentage within 7 days.

| **Variety** | **Treatment** | **Germination energy (%)** | **Germination rate (%)** |
| --- | --- | --- | --- |
| YN301 | 23d-T | 89.54±4.27 | 95.69±3.73 |
|  | 23d-CK | 1.67±2.89 | 5.56±5.09 |
|  | 27d-CK | 10.25±1.44 | 17.64±3.56 |
| YN1212 | 23d-T | 86.58±2.26 | 95.32±1.04 |
|  | 23d-CK | 9.81±3.41 | 19.61±6.81 |
|  | 27d-CK | 22.72±1.73 | 37.82±4.20 |
| KN9204 | 23d-T | 65.16±2.34 | 83.84±1.95 |
|  | 23d-CK | 3.33±2.89 | 6.85±2.74 |
|  | 27d-CK | 9.80±1.91 | 21.04±3.36 |
| JM22 | 23d-T | 86.75±2.36 | 94.73±2.15 |
|  | 23d-CK | 20.68±4.13 | 37.92±5.49 |
|  | 27d-CK | 44.44±5.09 | 57.56±8.44 |
| JM57 | 23d-T | 60.26±3.25 | 85.18±3.13 |
|  | 23d-CK | 1.33±2.31 | 2.62±2.27 |
|  | 27d-CK | 7.23±2.73 | 11.93±4.20 |
| ZM379 | 23d-T | 79.07±1.75 | 90.11±3.26 |
|  | 23d-CK | 23.96±4.71 | 33.95±5.19 |
|  | 27d-CK | 9.76±1.17 | 27.10±4.80 |
| ND753 | 23d-T | 62.79±4.61 | 85.48±5.60 |
|  | 23d-CK | 16.07±4.26 | 26.85±7.12 |
|  | 27d-CK | 5.96±2.07 | 12.05±1.86 |
| XN511 | 23d-T | 60.28±6.25 | 84.40±3.92 |
|  | 23d-CK | 2.52±2.18 | 6.83±2.80 |
|  | 27d-CK | 15.13±2.80 | 20.46±3.33 |
| XD33 | 23d-T | 77.91±1.85 | 90.16±3.88 |
|  | 23d-CK | 3.81±0.22 | 10.08±1.85 |
|  | 27d-CK | 3.20±2.81 | 10.63±2.36 |
| XM88 | 23d-T | 58.08±5.36 | 80.57±4.88 |
|  | 23d-CK | 3.75±3.31 | 20.51±6.57 |
|  | 27d-CK | 11.79±3.70 | 22.36±2.09 |

Note: 23d‑T: optimized treatment: harvest at 23 DAA, drying at 40 °C for 2 d, cold at 4 °C for 2 d. 23d‑CK: control at 23 DAA without further treatments. 27d‑CK: control at 27 DAA without further treatments. Raw data (total seeds, 3‑day and 7‑day germinated seeds per replicate) are available in Supplementary Data (Excel file, Sheet "Exp9_Variety_Raw").
